# Imaging small dynamic lesions using positron emission tomography and computed tomography: an ^18^F-sodium fluoride valvular phantom study

**DOI:** 10.1093/ehjimp/qyaf013

**Published:** 2025-02-28

**Authors:** Anna K Barton, Jacek Kwiecinski, Hidenobu Hashimoto, Mark Hyun, Keiichiro Kuronuma, Aditya Killekar, Aakriti Gupta, Nipun Manral, John Moore, Marc R Dweck, David E Newby, Daniel S Berman, Damini Dey, Piotr Slomka

**Affiliations:** Departments of Medicine (Division of Artificial Intelligence in Medicine) and Biomedical Sciences, Cedars-Sinai Medical Center, 6500 Wilshire Blvd, Los Angeles, CA 90048, USA; Centre for Cardiovascular Science, The University of Edinburgh, Edinburgh, UK; Departments of Medicine (Division of Artificial Intelligence in Medicine) and Biomedical Sciences, Cedars-Sinai Medical Center, 6500 Wilshire Blvd, Los Angeles, CA 90048, USA; Division of Cardiology and Structural Heart Diseases, Medical University of Silesia, Katowice, Poland; Departments of Medicine (Division of Artificial Intelligence in Medicine) and Biomedical Sciences, Cedars-Sinai Medical Center, 6500 Wilshire Blvd, Los Angeles, CA 90048, USA; Departments of Medicine (Division of Artificial Intelligence in Medicine) and Biomedical Sciences, Cedars-Sinai Medical Center, 6500 Wilshire Blvd, Los Angeles, CA 90048, USA; Departments of Medicine (Division of Artificial Intelligence in Medicine) and Biomedical Sciences, Cedars-Sinai Medical Center, 6500 Wilshire Blvd, Los Angeles, CA 90048, USA; Department of Cardiology, Nihon University, Tokyo, Japan; Departments of Medicine (Division of Artificial Intelligence in Medicine) and Biomedical Sciences, Cedars-Sinai Medical Center, 6500 Wilshire Blvd, Los Angeles, CA 90048, USA; Departments of Medicine (Division of Artificial Intelligence in Medicine) and Biomedical Sciences, Cedars-Sinai Medical Center, 6500 Wilshire Blvd, Los Angeles, CA 90048, USA; Departments of Medicine (Division of Artificial Intelligence in Medicine) and Biomedical Sciences, Cedars-Sinai Medical Center, 6500 Wilshire Blvd, Los Angeles, CA 90048, USA; Research and Development Division, Archetype BioMedical Inc., London, ON, Canada; Centre for Cardiovascular Science, The University of Edinburgh, Edinburgh, UK; Centre for Cardiovascular Science, The University of Edinburgh, Edinburgh, UK; Departments of Medicine (Division of Artificial Intelligence in Medicine) and Biomedical Sciences, Cedars-Sinai Medical Center, 6500 Wilshire Blvd, Los Angeles, CA 90048, USA; Departments of Medicine (Division of Artificial Intelligence in Medicine) and Biomedical Sciences, Cedars-Sinai Medical Center, 6500 Wilshire Blvd, Los Angeles, CA 90048, USA; Departments of Medicine (Division of Artificial Intelligence in Medicine) and Biomedical Sciences, Cedars-Sinai Medical Center, 6500 Wilshire Blvd, Los Angeles, CA 90048, USA

**Keywords:** positron emission tomography, cardiac phantom, cardiac computed tomography, multi-modality imaging

## Abstract

**Aims:**

^18^F-sodium fluoride (^18^F-NaF) positron emission tomography (PET) detects active microcalcification and predicts adverse outcomes including bioprosthetic valve deterioration. However, measuring small areas of ^18^F-NaF uptake within moving structures remains challenging, requiring further optimization. We developed a representative cardiac phantom to optimize ^18^F-NaF imaging of bioprosthetic valves.

**Methods and results:**

We placed a bioprosthetic valve with two pockets sutured to the leaflets mimicking valvular lesions and a subvalvular ring mimicking the valve remnant into the phantom and injected each with ^18^F-radionuclide (1 μCi pockets, 4 μCi ring). We injected the cardiac chambers with iohexol and ^18^F-radionuclide (0.176 mCi) for background activity. PET and computed tomography (CT) images were acquired using a Siemens Biograph Vision high-resolution digital PET/CT scanner. We analysed target-to-background ratio (TBR) and signal-to-noise ratio (SNR) and subjective measures of image quality. We compared results with a human case of transcatheter aortic valve replacement. Initially the SNR and TBR in the phantom greatly exceeded those from human imaging. We reduced the scan duration used for reconstruction to 30 and 15 s, achieving comparable results (30 s vs. 15 s vs. patient: SNR 45.6 vs. 13.9 vs. 44.3, TBR_max_ 6.5 vs. 5.4 vs. 4.1, noise 10.2% vs. 8.8% vs. 12.0%). With motion correction, SNR and image quality improved in the phantom (30 s 135.8 vs. 45.6, 15 s 32.9 vs. 13.9) but remained similar in the human case (47.3 vs. 44.3).

**Conclusion:**

A cardiac phantom can mimic clinical ^18^F-NaF valve bioprosthesis imaging, providing an opportunity to explore acquisition, reconstruction, and post-processing of ^18^F-NaF PET/CT for small mobile cardiac structures.

## Graphical Abstract

**Graphical Abstract:**
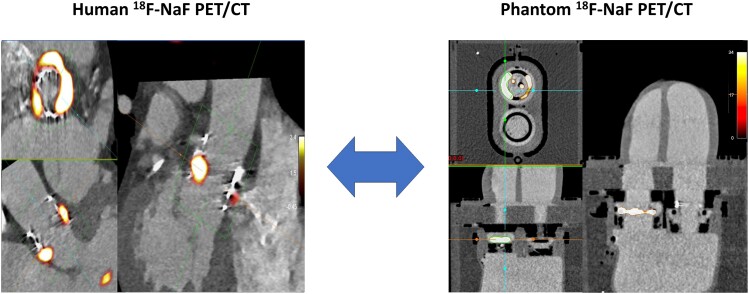

## Introduction

Positron emission tomography (PET) is an imaging modality that allows direct visualization of disease activity, subject to the availability of a radiolabelled tracer that targets the disease process of interest. Fluorine-18 labelled sodium fluoride (^18^F-NaF) is a radiotracer targeting active microcalcification.^1^ Its presence provides important prognostic information in many cardiovascular diseases in which calcification is a key driver, including coronary artery disease, aortic stenosis, and, most recently, degeneration and failure of aortic valve bioprostheses.^2–7^

Prosthetic valves are used to replace dysfunctional or damaged cardiac valves. This can be due to stenosis, where progressive calcification and narrowing of the valves impede the ejection of blood from or within the heart^8,9^; regurgitation, where blood leaks back due to failure of adequate valvular coaptation; or infection of the valvular apparatus due to infective endocarditis. A significant proportion of prosthetic valve replacements are implanted for stenosis of the aortic valve, a condition whose prevalence and severity increases with age.^10^

However, although it addresses the underlying pathology, valve replacements are not without their own complications. Bioprosthetic valve degeneration leading to failure is increasingly well-recognized.^3,4^ Factors predicting valvular failure are not completely understood, but calcification seems to be a key driver in their degeneration.^3,4,11,12^ In particular, as the population ages, there will be an increase in age-related conditions such as aortic stenosis where valve replacement remains the only treatment option.^10^ An increased prevalence of those with bioprosthetic valve replacements and therefore a larger population at risk of bioprosthetic degeneration and failure is anticipated. The ability to predict degeneration and future calcification with cardiovascular ^18^F-NaF PET imaging is therefore a highly relevant and important discovery in clinical cardiology.^3,4,11,12^

However despite these research advances, small, dynamic, and highly mobile structures, such as the heart valves, continue to present major technical challenges for PET imaging acquisition and analysis in clinical practice making accurate quantification of disease activity difficult.^13^ In addition, myocardial, respiratory, and patient motion can also degrade the PET signal quality.^13^

Phantoms are often used in optimization of imaging acquisition processes.^14–16^ The benefit of phantoms is the ability to repeat image acquisitions using different parameters on multiple occasions as unlike in human imaging, radiation burden and patient comfort and inconvenience are eliminated as limiting factors. Cardiovascular phantom models have been used effectively to investigate and to optimize PET image acquisition and reconstruction in nuclear cardiology, with some initial data concerning coronary imaging,^14–16^ but these studies did not include motion or valve imaging models.

In this work, we aimed to mimic *in vivo* human cardiovascular imaging conditions of degenerative bioprosthetic aortic valve disease using a bespoke representative cardiac phantom. Our goals were to optimize imaging acquisition, reconstruction, and post-processing protocols to image simulated degenerating valve bioprostheses using ^18^F-radionuclide PET and computed tomography (CT) imaging of our cardiac phantom.

## Materials and methods

### Cardiac phantom and accessories

**Figure 1:**
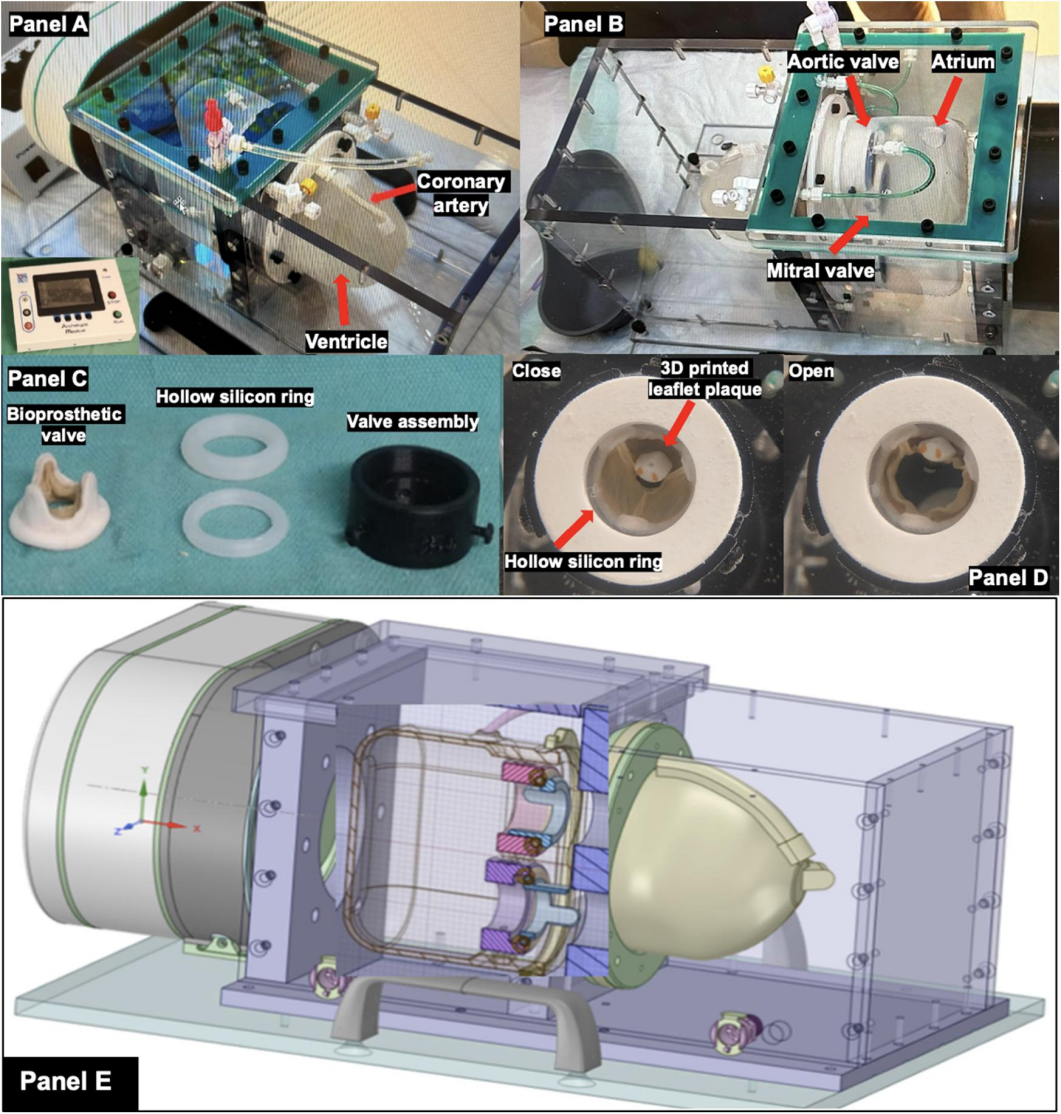
Our realistic cardiac phantom is displayed with imaging accessories to mimic bioprosthetic valve degeneration. (*A* and *B*) Photographs of our bespoke cardiac phantom displaying the different chambers and components, as well as the transparent polycarbonate box that contains it and the electronic module that allows variation of the contraction rate and force. (*C*) A picture of bioprosthetic aortic valve, hollow silicon ring, and valve assembly. (*D*) A 3D-printed plaque with pockets onto a bioprosthetic valve leaflet to mimic degeneration and allow radiotracer infiltration for PET imaging sessions. (*E*) A technical drawing of the phantom.

We developed our cardiac phantom in collaboration with Archetype BioMedical Inc. (London, Canada) who produced the final product according to our specifications and requirements. With two different models comprised of silicon or polyvinyl alcohol cryogel, respectively, the phantom consists of four chambers mimicking the left atrium, two ventricles, and proximal aorta and two valve modules in the mitral and aortic position (*Figure 1*). The ventricles contract in response to the device control panel which can be adjusted to reflect a range of physiological human heart rates. A customized pulsatile flow pump stationed adjacent to the phantom in a watertight container simulates blood flow through the chambers and thus passive motion of the valvular structures as occurs *in vivo*.

To allow us to simulate regions of radiotracer uptake, 3D-printed silicon modules were also developed by Archetype BioMedical in order to incorporate one small (0.04 mL) and one large (0.12 mL) pocket on the valve bioprosthetic leaflets, and a hollow ring (0.5 mL) that was placed in the subvalvular position to mimic disease activity from the remnant of the native aortic valve as seen in patients who undergo valve replacement (*Figure 1*).

We utilized two Medtronic Mosaic tissue valves (Medtronic, Minneapolis, MN, USA). The two pocket modules were sutured with care to valve leaflets on one of the Medtronic Mosaic bioprostheses to mimic tracer-avid lesions often observed on degenerating aortic bioprostheses (*Figure 1*).

### Image acquisition

**Figure 2:**
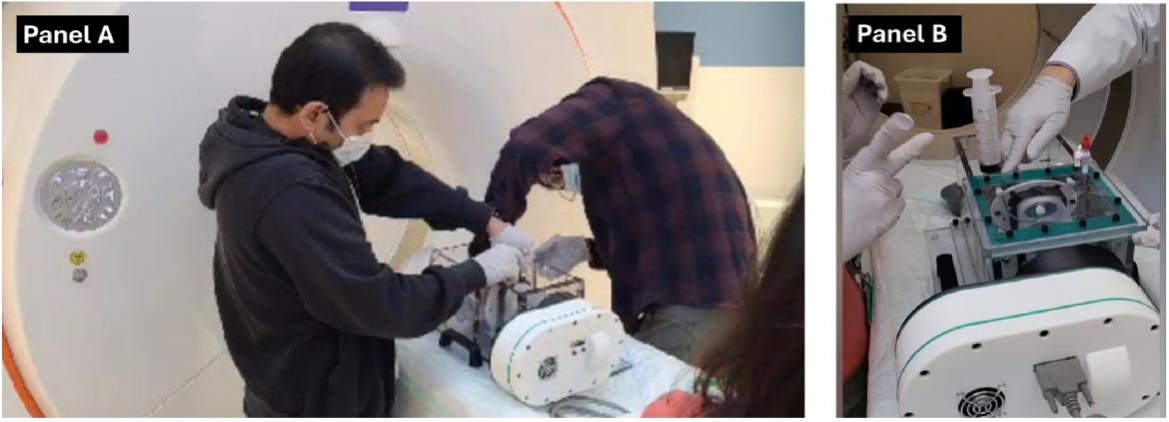
Preparation of the cardiac phantom for hybrid PET/CT imaging (*A*). Following the activity injection into the dedicated pockets, the chamber is filled with 700 mL of 0.9% sterile sodium chloride and 50 mL iodinated contrast (iohexol 350 mg iodine/mL, Omnipaque 350™) (*B*). Subsequently, the phantom is ready for scanning and emission scanning commences.

All imaging was performed at Cedars-Sinai Medical Center, Los Angeles, on a Siemens Biograph Vision high-resolution digital PET/CT scanner (Siemens Healthcare, Germany) (*Figure 2*). In total, we performed five phantom imaging acquisitions using either contrast-enhanced cardiac CT (CCT, *n* = 3) or PET/CT (*n* = 2). Our objectives were as follows: (i) to establish appropriate iohexol volume to maintain a 350–500 Hounsfield units (HU) attenuation within the heart and aorta for optimal valve bioprosthesis visualization on the CCT acquisition, (ii) to isolate the atrial chamber from the transparent polycarbonate box containing the phantom, and (iii) to optimize physiological parameters and gating for CCT and attenuation correction sequence. The final two sessions also included a PET acquisition. Here, we aimed to determine tracer dosage to mimic (i) regions of interest (ROIs) of a degenerating aortic valve bioprosthesis and (ii) background activity.

### CT acquisition and reconstruction

Initially, CT attenuation correction of the phantom was performed with a craniocaudal acquisition of 1-mm slice thickness over a large (780-mm) field of view (FoV). Following this, a prospectively gated axial CCT was acquired from the cranial to the caudal position with thin (1-mm) slice thickness over a 260-mm FoV. All acquisitions used a tube voltage of 120 kVp. CCT was acquired using prospective gating over 10 gates of the simulated cardiac cycle. Optimal physiological conditions in human imaging were mimicked by a simulated ventricular contraction rate of 60 bpm which provided excellent image quality with precise characterization of the aortic bioprosthesis at all stages of the cardiac cycle.

An iterative reconstruction algorithm (sinogram affirmed iterative reconstruction; SAFIRE)^17^ was then applied to the 10-gate CCT sequence obtained with soft tissue (Br38) kernels. Reconstructions of PET data were all performed using dedicated reconstruction from Siemens. The PET matrix size was 440 × 440 × 524. Gaussian post-filtering (2 mm SD) was applied using FusionQuant software (FusionQuant, Cedars-Sinai Medical Center, Los Angeles)^18^ with pixel size 0.83 × 0.83 × 0.5 mm.

### CT contrast density determination

**Figure 3:**
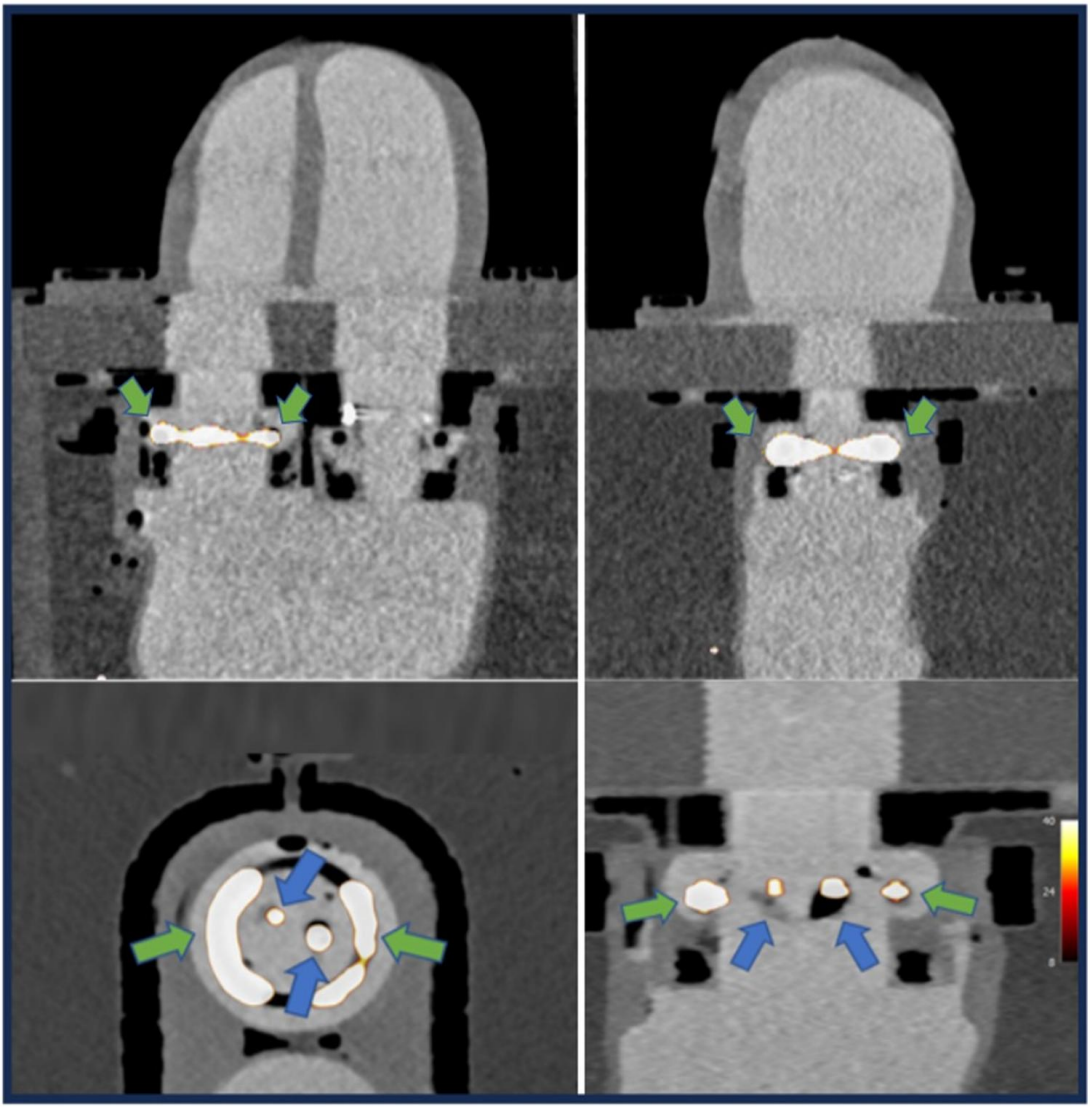
Hybrid PET/CCT acquisition of the entire silicon cardiac phantom model in horizontal and vertical long axes (top row), with short- and long-axis views focusing on the degenerative aortic valve module (bottom row). Tracer activity representative of remnant native valve disease (green arrows) and leaflet degeneration (blue arrows) is displayed.

We filled the phantom with 700 mL of 0.9% sterile sodium chloride and introduced iodinated contrast agent (iohexol 350 mg iodine/mL, Omnipaque 350™) in 25 mL increments. Administration of 50 mL iohexol 350 mg iodine/mL achieved a cardiac chamber attenuation of 350–500 HU providing excellent visualization of the aortic valve bioprosthesis and the ventricular walls (*Figure 3*). These concentrations were maintained for the duration of image acquisition, unlike in human imaging when contrast quickly dissipates throughout the circulating volume.

### PET radiotracer

Because our *ex vivo* phantom model incorporating aortic valve bioprostheses does not feature structural abnormalities, which are present in the aortic valve disease process of interest, we substitute these with hollow modules into which a radiotracer can be injected. Therefore, any tracer can be used with mutually applicable results provided it is radiolabelled identically to the tracer of interest. For this reason, we used excess clinical doses of ^18^F-fluorodeoxyglucose (^18^F-FDG) in our phantom experiments in place of ^18^F-NaF.

### PET acquisition and reconstruction

In acquisitions where PET was included, PET images were acquired following acquisition of CT attenuation correction and CCT as described above. The injected dose to the phantom was corrected for the decay. A 1 μCi ^18^F-FDG was injected into each of the larger (0.12 mL) and smaller (0.04 mL) pockets on the bioprosthesis leaflets. The ring on the periphery of the valve was filled with 4 μCi (0.5 mL) of ^18^F-FDG. We injected 0.176 mCi of ^18^F-FDG into the 750 mL heart chambers to mimic background activity seen in *in vivo* imaging. This also allowed us to assess noise and the signal-to-noise ratio (SNR). A 10-min list-mode PET acquisition was performed 10 min following injection of the radiotracer to whichever module that was injected first (*Figure 2*). This allowed for several independent 15-s reconstructions. The data set was reconstructed using an ultra-high definition algorithm with time of flight on a 440 × 440 matrix (slice thickness 0.8 mm, pixel spacing 0.8 mm) using four iterations and five subsets. Rather than embedding Gaussian filtering within the reconstruction, we performed it post-reconstruction in a dedicated software package.

### PET cardiac motion correction

Motion correction was performed to align PET images from all 10 cardiac gates preserving all counts. This was done using a mass-preserving, diffeomorphic, anatomically guided Demons method to optimize global energy between PET frames. In the initial step, we created a sphere encompassing the ROI labelled as a valvular ROI. We utilized a non-linear registration algorithm with radial constraint to align PET data to the diastolic gate. PET signal [maximum target-to-background ratio (TBR_max_), SNR] was quantified before and after the application of motion correction.^19–21^

### Image analysis

Analysis of PET imaging was performed using FusionQuant. A ROI was drawn over each of the three bioprosthesis ROIs into which the ^18^F-labelled tracer had been administered, namely, the two silicon pockets sutured to the bioprosthesis leaflets and the subvalvular ring. A 2-mm 3D Gaussian filtering was performed within FusionQuant. We then measured the maximum standardized uptake value (SUV_max_) for each ROI. These values were corrected for mean blood pool activity obtained from the atrial chamber to give a maximum TBR (TBR_max_). Image noise was calculated by dividing bloodpool SUV_max_ by the standard deviation of the bloodpool SUV_mean_. The SNR was then calculated by dividing the maximum uptake in each ROI by the image noise. The two valvular lesions were measured using a 4 (small) and 5 (large) mm sphere, and the native valve uptake was assessed using a 4-mm centreline around the circumference of the valve.

### Human comparison of bioprosthetic dysfunction disease

To provide a similar clinical comparator for our phantom imaging acquisitions, we performed a repeat analysis of a clinical case of transcatheter aortic valve replacement (TAVR) dysfunction from the RESOLVE study.^22^ The patient was imaged for 30 min commencing 1 h after administration of 3.4 mCi of ^18^F-NaF. This case was obtained as part of a separate research study, and the participants provided full informed consent in line with the study’s ethical approval.^22^

## Results

### PET image reconstruction and quantification

#### Reconstruction

Despite using low tracer activities, initial reconstructions using 5 min of list-mode data gave excellent PET signal with TBR values measured on the diastolic gates (gate 7 of 10) markedly higher than those of clinical bioprostheses ^18^F-NaF imaging (TBR_max_ 11.5 vs. 6.7). The duration of PET reconstruction was therefore reduced to 30 and then 15 s to match SNR seen in human imaging, and the same Gaussian post-filtering was applied. For the human TAVR bioprosthetic case, Gaussian filtering was already applied in the reconstruction and therefore was not repeated.

### Quantification of PET signal

**Figure 4:**
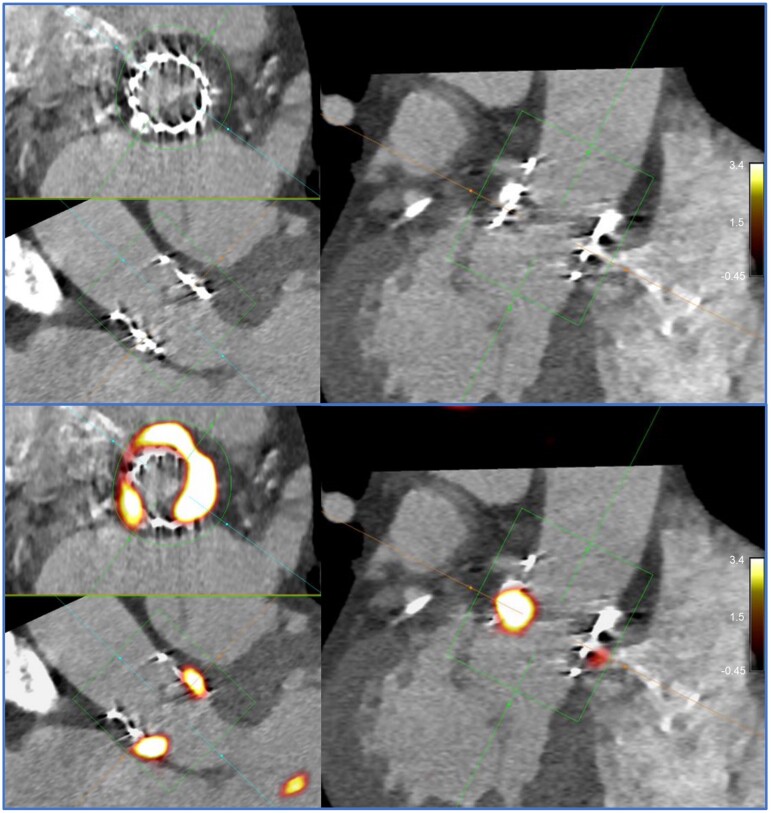
Hybrid ^18^F-NaF PET/CT acquisition in a patient with known TAVR degeneration. Intense tracer signal is seen localized to the TAVR, in particular the position of the prior non-coronary cusp of the aortic valve. In this case, TBR_max_ was calculated at 4.1 (SUV_max_ value 5.7, mean left atrial blood pool 1.4±0.1) and SNR was 47.3 after application of motion correction.

With a shortened PET reconstruction time of 30 s, we achieved quantifiable valvular PET signal similar to human TAVR imaging (*Table 1* and *Figure 3*). This included SNR (45.6 vs. 44.3) and TBR_max_ (6.5 vs. 4.1) for the small valvular ROIs compared with the human case. Background noise within the cardiac chambers (10.2% vs. 12.0%) was also comparable. Other areas of interest within the phantom (large pocket and ring lesion) also showed high PET signal (*Table 1* and *Figure 3*), markedly exceeding the comparable human case even with the very short (30 s) reconstruction time (*Figure 4*). We then reduced the duration of reconstructed PET data to 15 s. This yielded signal for both small (TBR_max_ 5.4) and large (TBR_max_ 6.0) valvular ROIs that was more comparable with human imaging (TBR_max_ 4.1), though SNR for both ROIs (small pocket 13.9, large pocket 15.6) was lower than in the clinical case of TAVR dysfunction (44.3).

**Table 1:** Analysis of 30 s PET/CT acquisition of our cardiac phantom alongside values from a comparable human case of TAVR dysfunction also imaged using ^18^F-labelled radiotracer

	Cardiac chambers	Native valve remnant (30-s recon)	Native valve remnant (15-s recon)	Valvular lesions (30-s recon)	Valvular lesions (15-s recon)	Human TAVR lesion
Volume	750 mL	0.5 mL	0.5 mL	Large: 0.12 mL Small: 0.04 mL	Large: 0.12 mL Small: 0.04 mL	.
Iohexol 350 mg i/mL	50 mL	.	.	.	.	.
^18^F-tracer activity	0.176 mCi	4 µCi	4 µCi	Large: 1 μCi Small: 1 μCi	Large: 1 μCi Small: 1 μCi	3.4 mCi
TBR_max_	.	21.9	19.9	Large: 10.2 Small: 6.5	Large: 6.0 Small: 5.4	4.1
SNR	.	153	51.3	Large: 71.1 Small: 45.6	Large: 15.6 Small: 13.9	44.3
SNR (motion corrected)	.	.	154.3	Small: 135.8	Large: 35.8 Small: 32.9	47.3
Noise (background)	.	10.2%	8.8%	10.2%	8.8%	12.0%

Using these reconstructive parameters, comparable values of tracer expression were achieved, with good agreement in background activity and image noise. HUs within the phantom reached levels desirable for human imaging (300–350) using a 1:14 concentration of iohexol contrast agent relative to sterile 0.9% sodium chloride solution.

### Application of motion correction

**Figure 5:**
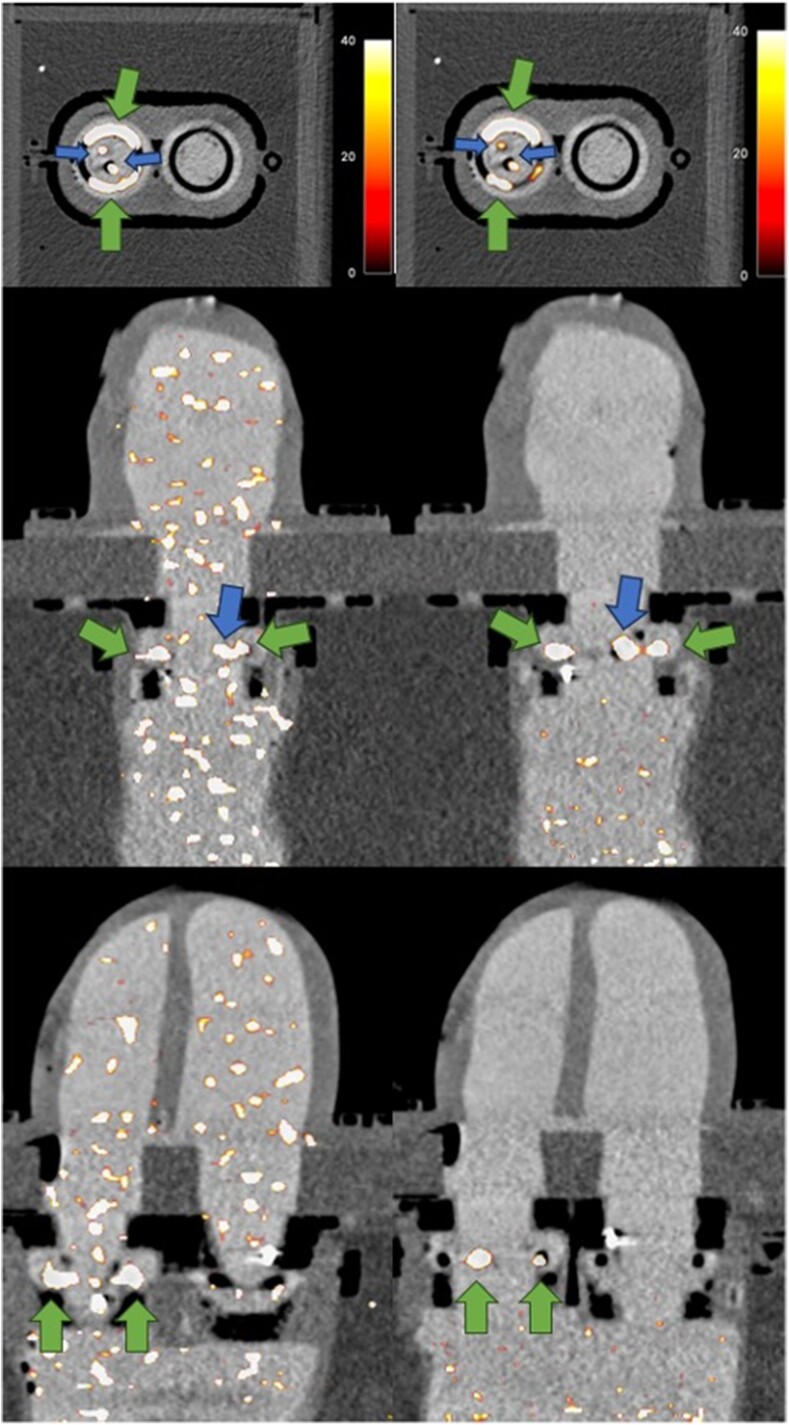
Image quality before (left) and after application of valvular motion correction is displayed. From top to bottom, a short-axis view through the valves and vertical and horizontal axis views through the atrial and ventricular chambers are shown. Alongside subjective improvement in image quality, superior SNRs were achieved in the native valve remnant (green, larger arrows) and valvular lesions (blue, smaller arrows) with motion correction applied.

Following motion correction of the PET data, we observed further improvements in SNR in the valvular small pocket relative to the non-motion corrected phantom acquisition (30 s reconstruction: 135.8 vs. 45.6, 15 s recon 32.9 vs. 13.9) and subjective image quality (*Table 1* and *Figure 5*). In contrast for the human TAVR case, SNR was essentially unchanged following application of motion correction (47.3 vs. 44.3).

## Discussion

To the best of our knowledge, this study is the first to test the utility of a realistic cardiac phantom for PET/CT imaging of bioprosthetic valve degeneration. Advanced cardiac phantoms provide an excellent opportunity to optimize the acquisition, reconstruction, and post-processing of ^18^F-NaF digital PET/CT assessments of small moving structures, as multiple repeated acquisitions with different tracer activity ratios can be performed.^13,14^ However, previous cardiovascular phantoms did not allow imaging of small moving structures, such as cardiac valves, with previous work focusing instead on imaging of the myocardium rather than the valves.^15^ This work proves the concept of a realistic cardiac phantom in optimizing PET image acquisition, reconstruction, and analysis in small, thin, highly mobile structures within the cardiovascular system. By employing a realistic phantom, we have established effective parameters for imaging valvular prostheses with simulated degeneration using PET/CT.

^18^F-NaF PET is an established technique that provides a non-invasive assessment of calcification activity within the bones and cardiovascular system.^6,23,24^ A major recent development is the demonstration of its ability to detect degeneration of aortic valve prostheses.^3,4,11^ Our findings are likely to be of significant utility given the rising number of bioprosthetic aortic valve replacements being performed worldwide, leading to a growing population in whom bioprosthetic valve failure is a possibility. Identifying those at increased risk for additional surveillance emerges as a critical clinical need, and ^18^F-NaF PET has the potential to address this need and become a vital tool in optimizing the management of this complex and growing patient group. Many other ^18^F-labelled PET tracers provide pathological insights in cardiovascular disease, such as thrombus formation in both the coronaries and cardiac valves (^18^F-GP1),^25,26^ and myocardial inflammation and viability assessments (^18^F-fluorodeoxyglucose).^27,28^ Our findings are, therefore, additionally likely to be useful in optimizing PET assessments in these settings.

Our use of very small valvular lesion sizes with minimum volumes of only 0.04 mL mimics the very small areas of valvular degeneration as can be seen in human disease in earlier stages, as well as larger lesions (0.12 mL) and the native valve remnant, making ours a highly novel and accurate model for aortic valve prosthetic dysfunction. Our parameters are likely to inform optimal techniques for clinical imaging of patients with degenerative aortic valve prostheses. In particular, we found that our phantom required very low levels of tracer activity with very short reconstruction lengths to achieve values comparable with patient imaging of the same condition. A natural next step would be imaging a large clinical cohort with degenerative aortic valvular prostheses using PET/CT with image acquisition and reconstruction parameters informed by our phantom experiments.

### Limitations

While our phantom provides excellent simulation of cardiac motion caused by myocardial contraction and intracardiac blood flow, it does not simulate respiratory patient motion, another important factor that can impede accurate quantification of PET activity.^13^ We performed imaging on a single PET/CT scanner which may limit the generalizability of our findings to other makes and models. Future work should investigate different scanners for confirmation. Finally, although extreme care was taken to mimic as closely as possible human degenerative bioprosthetic valve disease and actual valve implants were used, there will always be differences between even the most sophisticated of phantoms and the unparalleled complexity of human pathology.

## Conclusions

We utilized a realistic heart phantom to explore and successfully mimic clinical imaging conditions of aortic valve bioprosthetic dysfunction using hybrid ^18^F-NaF PET/CT.

## Consent

The patient case in this study was obtained as part of a separate research study (RESOLVE study), and the participants provided full informed consent in line with the study’s ethical approval.

## Data Availability

To the extent allowed by data sharing agreements and institutional review board protocols, the data from this manuscript will be shared upon written request.

## Lead author biography

Dr Anna Barton is a specialty trainee in cardiology in NHS Lothian, Scotland. She received her primary medical degree and Master of Science from the University of Edinburgh, where she is currently a PhD student. Dr Barton’s clinical interests include heart failure, multi-modality cardiac imaging, and preventative cardiology. Her research work focuses on the role of novel PET tracers in cardiovascular disease.
